# *RGS1* and *CREB5* are direct and common transcriptional targets of ZNF384‐fusion proteins

**DOI:** 10.1002/cam4.7471

**Published:** 2024-07-17

**Authors:** Chiharu Yamada, Kentaro Okada, Koya Odaira, Mahiru Tokoro, Eisuke Iwamoto, Masashi Sanada, Mina Noura, Syuichi Okamoto, Takahiko Yasuda, Shinobu Tsuzuki, Hitoshi Kiyoi, Fumihiko Hayakawa

**Affiliations:** ^1^ Division of Cellular and Genetic Sciences, Department of Integrated Health Sciences Nagoya University Graduate School of Medicine Nagoya Japan; ^2^ Clinical Research Center National Hospital Organization Nagoya Medical Center Nagoya Japan; ^3^ Department of Biochemistry Aichi Medical University School of Medicine Nagakute Japan; ^4^ Department of Hematology and Oncology Nagoya University Graduate School of Medicine Nagoya Japan

**Keywords:** acute lymphoblastic leukemia, fusion gene, RNA‐seq, transcriptional target, *ZNF384*

## Abstract

**Background:**

ZNF384‐fusion (Z‐fusion) genes were recently identified in B‐cell acute lymphoblastic leukemia (B‐ALL) and are frequent in Japanese adult patients. The frequency is about 20% in those with Philadelphia chromosome‐negative B‐ALL. ZNF384 is a transcription factor and Z‐fusion proteins have increased transcriptional activity; however, the detailed mechanisms of leukemogenesis of Z‐fusion proteins have yet to be clarified.

**Methods:**

We established three transfectants of cell lines expressing different types of Z‐fusion proteins, and analyzed their gene expression profile (GEP) by RNA‐seq. We also analyzed the GEP of clinical ALL samples using our previous RNA‐seq data of 323 Japanese ALL patients. We selected upregulated genes in both Z‐fusion gene‐expressing transfectants and Z‐fusion gene‐positive ALL samples, and investigated the binding of Z‐fusion proteins to regulatory regions of the candidate genes by ChIP‐qPCR.

**Results:**

We selected six commonly upregulated genes. After the investigation by ChIP‐qPCR, we finally identified *CREB5* and *RGS1* as direct and common target genes. RGS1 is an inhibitor of CXCL12‐CXCR4 signaling that is required for the homing of hematopoietic progenitor cells to the bone marrow microenvironment and development of B cells. Consistent with this, Z‐fusion gene transfectants showed impaired migration toward CXCL12.

**Conclusions:**

We identified *CREB5* and *RGS1* as direct and common transcriptional targets of Z‐fusion proteins. The present results provide novel insight into the aberrant transcriptional regulation by Z‐fusion proteins.

## Graphical abstract

ZNF384‐fusion genes were recently identified in B‐cell acute lymphoblastic leukemia (B‐ALL) and are frequent in Japanese adult patients. We searched for direct transcriptional targets of ZNF384‐fusion proteins by analyzing the gene expression profile of Z‐fusion gene‐transfected cell lines and clinical ALL samples. We identified CREB5 and RGS1 as direct and common transcriptional targets of ZNF384‐fusion proteins.

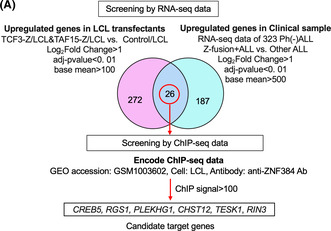

## 1 INTRODUCTION

The zinc finger protein 384 (ZNF384) is located on human chromosome 12p13.31. ZNF384 is a transcription factor that binds to promoters through the consensus DNA sequence (G/C)AAAAA(A) and was originally identified as a regulator of extracellular matrix genes, such as matrix metalloproteinases (MMPs) 1, 3, and 7 and the type I collagen a 1 polypeptide chain (COL1A1).^1^, ^2^ Gene‐targeting experiments in mice also indicated roles of ZNF384 in bone metabolism^3^ and spermatogenesis.^4^ ZNF384 forms fusion genes with various partner genes, numbering more than 10, such as E1A‐binding protein P300 (EP300), transcription factor 3 (TCF3), and TATA box‐binding protein‐associated factor 15 (TAF15).^5^, ^6^, ^7^ Z‐fusion genes are the most frequent fusion genes in Japanese adult *BCR::ABL1*‐negative B‐cell acute lymphoblastic leukemia (B‐ALL), while they are much less frequent in the US. The frequencies are 23 and 3%, respectively.^8^, ^9^ They are also frequent in China and Italy, comprising 19% and 9% of B‐ALL without major genetic abnormalities such as *BCR::ABL1, TCF3::PBX1, ETV6::RUNX1*, or *KMT2A* rearrangement,^10^, ^11^ respectively, suggesting that they are tend to be more frequent in Asian patients. We and others demonstrated the development of leukemia by introducing a Z‐fusion gene into mouse hematopoietic cells, strongly indicating its leukemogenicity.^12^, ^13^, ^14^ We also found that Z‐fusion proteins showed high affinity for EP300 and increased transcriptional activities^15^; however, detailed mechanisms of leukemogenesis of Z‐fusion proteins, such as direct and common transcriptional targets of Z‐fusion proteins, and how those genes contribute to leukemia development have yet to be clarified.

In the present study, we searched for direct transcriptional targets of Z‐fusion proteins by analyzing the gene expression profile of Z‐fusion gene‐transfected cell lines and clinical ALL samples. We identified *CREB5* and *RGS1* as direct and common transcriptional targets of Z‐fusion proteins. The present results provide novel insight into the aberrant transcriptional regulation by Z‐fusion proteins.

## 2 MATERIALS AND METHODS

### 2.1 Cell culture

HEK293 and HEK293T were cultured in Dulbecco's modified Eagle's medium (DMEM, Wako, Osaka, Japan) supplemented with 10% FBS (Thermo Fisher Scientific). LCL, comprising human mature B cells transformed by the Epstein–Barr virus, and NALM‐6, human *DUX4::IGH*‐positive B‐ALL cell line, were cultured in Roswell Park Memorial Institute medium 1640 (RPMI‐1640, Wako) supplemented with 10% FBS. JIH‐5, a human *EP300::ZNF384* (*EP300‐Z*)‐positive ALL cell line, was cultured in Iscove's Modified Dulbecco's Medium (IMDM, Wako) supplemented with 20% FBS.

### 2.2 Plasmid construction

Fragments of cDNA of *TCF3::ZNF384* (*TCF3‐Z*) and *TAF15::ZNF384* (*TAF15‐Z*) were amplified by PCR from cDNA samples of *TCF3‐Z*‐positive ALL and *TAF15‐Z*‐positive ALL, respectively under the study approved by the Institutional Review Board, the ethics committee of Nagoya University Graduate School of Medicine. Blood samples were collected from patients with written informed consent. They were inserted into N‐Flag/pcDNA at sites of EcoRI and NotI using the In‐Fusion HD Cloning Kit (Takara Bio, Shiga, Japan) to create Flag‐TCF3‐Z/pcDNA and Flag‐TAF15‐Z/pcDNA, respectively. Flag‐ZNF384/pcDNA, FLAG‐EP300‐Z/pcDNA, and Flag‐EP300‐Z/pCMV were described previously.^15^ FLAG‐TCF3‐Z/pCSII, FLAG‐TAF15‐Z/pCSII, and FLAG‐EP300‐Z/pCSII were constructed by the insertion of cDNA fragments of Flag‐Z‐fusion genes cut out from corresponding N‐Flag/pcDNA vectors at BamHI site and blunt‐ended NotI site into pCSII_EFI Tet‐on ires GFP at BamHI and HpaI sites. pCSII_EFI Tet‐on ires GFP is a lentivirus vector expressing a gene of interest in a doxycycline (Dox)‐dependent manner.^16^ ZNF384 cDNA was cut out from Flag‐ZNF384/pcDNA at EcoRI and NotI sites and inserted into Myc/pSG5 at the same sites to form Myc‐ZNF384/pSG5. RGS1/pCMV6‐Entry (RC218112) and CREB5/pCMV6‐Entry (RC213568) were purchased from ORIGENE (Rockville, MD, USA). Luciferase reporter genes, RGS1‐pr/pGL4 and CREB5‐pr/pGL4, were made by the insertion of the regions from −497 to −77 and from +6 to +174 from the transcription start site of RGS1 and CREB5, respectively, into pGL4 (Promega, Madison, WI, USA).

### 2.3 Antibodies

The anti‐ZNF384 antibody (ab251673) was purchased from Abcam plc. (Cambridge, UK). RGS1 antibody N‐term (AP8758a‐ev) and CREB5 antibody N‐term (AP14245a‐ev) were from Abcepta (Suzhou, China). The anti‐beta‐actin antibody (3598R) was obtained from BioVision Inc. (Milpitas, CA, USA).

### 2.4 Knockdown experiments and cell growth assay

The expression vectors of shRNA were made by the insertion of shRNA sequences to pS65G. shRNA for RGS1 and CREB5 were designed according to previous reports^17^, ^18^ and targeted 5′‐TGAATGAGTGGTTCCTTTC‐3′ and 5′‐CAGTATTCTGTAGGATCTA‐3′, respectively. pS65G expressed GFP as a transduction marker in addition to shRNA, as described previously.^19^ These vectors were introduced into JIH5 with a super electroporator, NEPA21 (NEPAGENE, Ichikawa, Japan). The rate of GFP‐positive live cells was measured by FACSCalibur (BD Biosciences, Franklin lakes, NJ, USA).

### 2.5 Cell migration assay

The cell migration assay was performed with Cytoselect™ Cell Migration Assay (Cell Biolabs Inc., San Diego, CA, USA), according to the manufacturer's instructions.

### 2.6 Transient gene introduction, lentivirus infection, immunoblot, luciferase assay, reverse transcription‐quantitative polymerase chain reaction (RT‐qPCR), RNA‐seq, and chromatin immunoprecipitation

These experiments were performed as described previously.^16^ The primers used in qPCR are shown in Supplemental Table S1.

## 3 RESULTS

### 3.1 Establishment of stable transfectants of Z‐fusion genes

**FIGURE 1:**
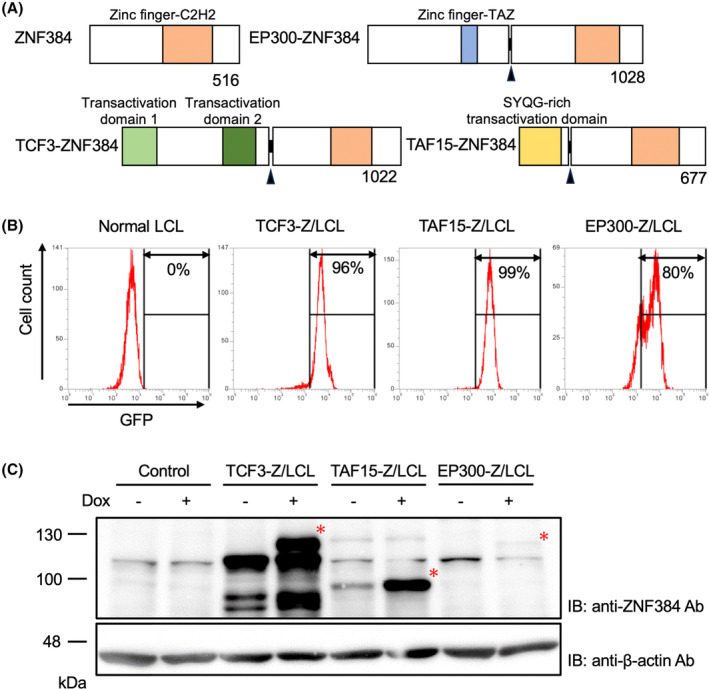
Establishment of LCL transfectants. (A) Schematic presentation of the structures of Z‐fusion proteins. Arrowheads indicate break points of the fusion proteins. (B) Histograms of GFP‐expressions in LCL transfectants. GFP‐expressions of representative clones after limiting dilution were analyzed by frow cytometry. (C) Dox‐dependent expression of Z‐fusion proteins in LCL transfectants. The indicated cells were cultured with or without 1 μg/mL Dox for 24 h as indicated, lysed, and subjected to SDS‐PAGE following immunoblot with the indicated antibodies. Asterisks indicate the position of Z‐fusion proteins.

We lentivirally transduced Dox‐dependent expression vectors of the three most frequent Z‐fusion proteins, EP300::ZNF384 (EP300‐Z), TCF3‐Z, and TAF15‐Z, into LCL. Their protein structures are shown in Figure 1A. After viral transfection, the transfected cells were collected by sorting of GFP‐positive cells. Then, mono or oligo clones were obtained by the limiting dilution method. Clones with the strongest expression of Z‐fusion proteins were selected for further experiments and designated as EP300‐Z/LCL, TCF3‐Z/LCL, and TAF15‐Z/LCL, respectively. Control LCL, a stable transfectant of the empty vector, was also established by the same method. GFP expression and Dox‐dependent expressions of Z‐fusion proteins in the representative clones are shown in Figure 1B,C. The GFP expression of EP300‐Z/LCL had two peaks, indicating that this clone contained at least two clones. In addition, Z‐fusion protein expression in EP300‐Z/LCL was weak compared with the other transfectants; therefore, we excluded this transfectant from further experiments.

### 3.2 Search for transcriptional target genes of Z‐fusion proteins

**FIGURE 2:**
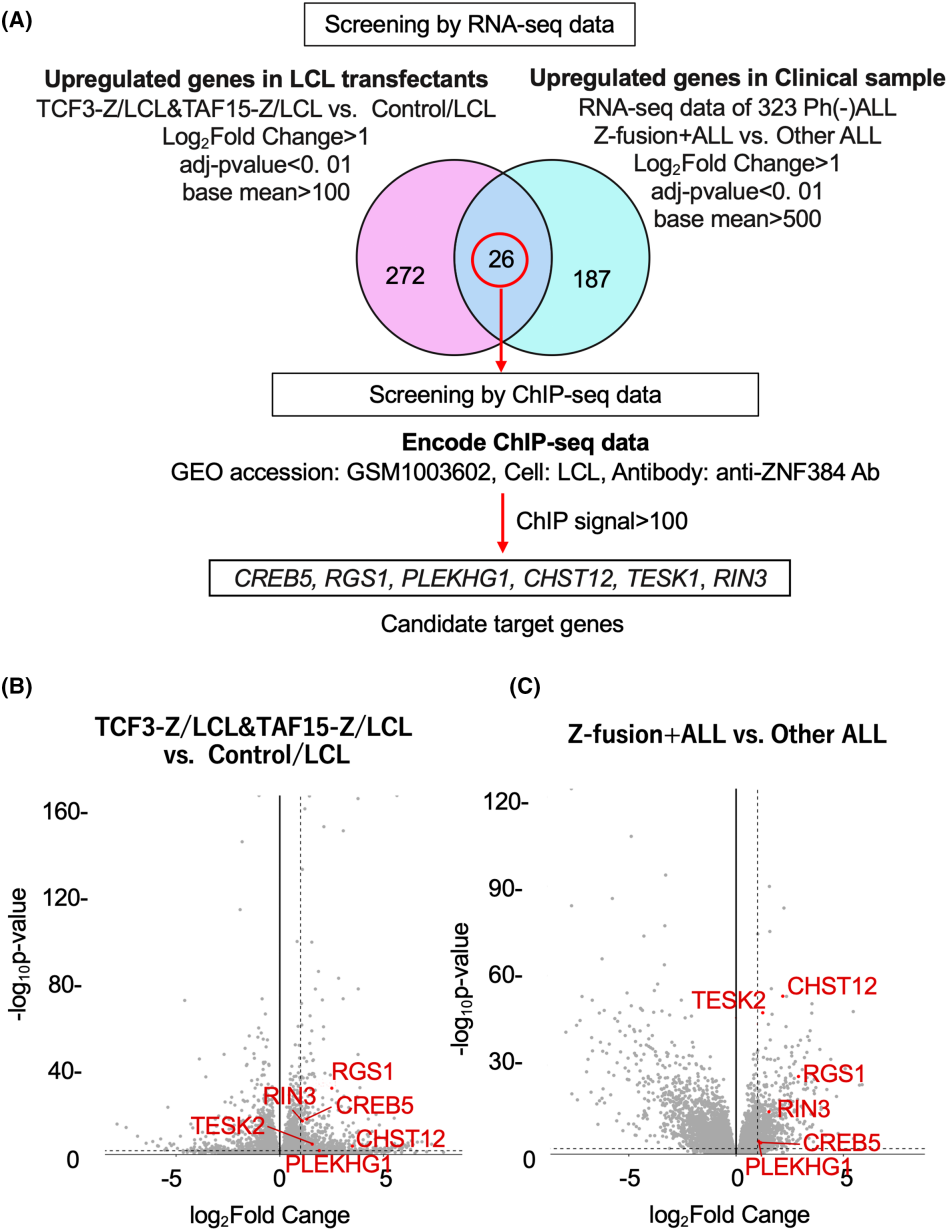
Search for transcriptional target genes of Z‐fusion proteins. (A) A flowchart of screening to identify direct and common transcriptional target genes of Z‐fusion proteins is shown. Volcano plots of gene expression in LCL transfectants (B) and clinical samples (C) The six candidate genes are plotted in red. The vertical dashed lines indicate Log_2_Fold Change = 1. The horizontal dashed lines indicate adjusted −Log_10_ *p*‐value = 2.

A flowchart of the screening to identify direct and common transcriptional target genes of Z‐fusion proteins is shown in Figure 2A. We performed RNA‐seq and analyzed differentially expressed genes (DEG) between the Z‐fusion gene‐transfectant and the control transfectant. We searched for genes commonly upregulated in Z‐fusion gene‐transfectants, and selected 272 genes satisfying the following conditions: log_2_ Fold Change >1, adjusted *p*‐value < 0.01, and base mean > 100 (Supplemental Table S2). Next, we similarly analyzed our previous RNA‐seq data of 323 Japanese Philadelphia chromosome‐negative adult B‐ALL samples containing 79 Z‐fusion gene‐positive ALL (Z‐fusion (+) ALL) to identify genes upregulated in Z‐fusion (+) ALL compared with other ALL. We selected 187 genes satisfying the following conditions: log_2_ Fold Change >1, adjusted *p*‐value < 0.01, and base mean > 500 (Supplemental Table S3). Out of 26 overlapping genes, we selected six genes bound by ZNF384 using ENCODE ChIP‐seq data (GEO accession: GSM1003602).^20^ The candidate genes were: *CREB5*, *RGS1*, *RIN3*, *PLEKHG1*, *CHST12*, and *TESK2*. The positions of the candidate genes in volcano plots are shown in Figure 2A,B.

### 3.3 CREB5 and RGS1 were direct transcriptional targets of both TCF3‐Z and TAF15‐Z

**FIGURE 3:**
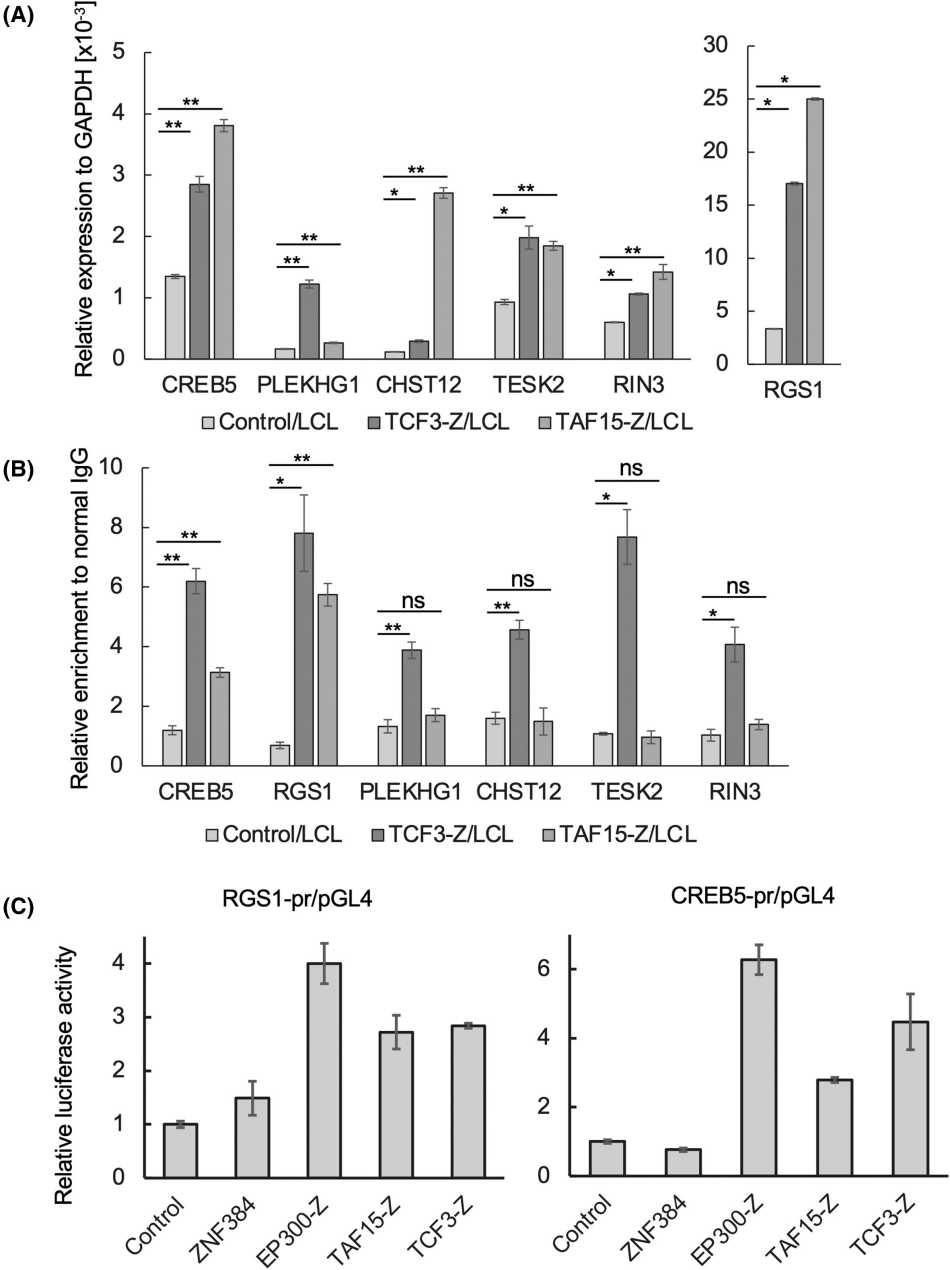
*CREB5* and *RGS1* were direct transcriptional targets of both TCF3‐Z and TAF15‐Z. (A) Upregulations of the candidate genes in LCL transfectants. mRNA was extracted from the indicated cells after culture with 1 μg/mL Dox for 24 h and subjected to RT‐qPCR. Expression levels of the indicated genes are plotted on bar charts as values relative to *GAPDH* expression. Experiments were independently performed three times in duplicate, and average values were plotted. Error bars indicate the standard error of the mean (SEM). Welch's *t*‐test was used for the statistical test. *p < 0.05; **p < 0.01; ns, Not significant. (B) Binding of Z‐fusion proteins to the candidate genes in LCL transfectants. ChIP‐qPCR was performed using the indicated cells and anti‐Flag antibody. Enrichments of the target DNA fragments by anti‐Flag antibody relative to that by normal IgG are plotted on bar charts. Experiments were independently performed three times in duplicate and average values were plotted. Error bars indicate SEM. Statistical analyses were done as in (A). (C) Z‐fusion proteins showed transcriptional activity for the reporter gene using the promoter elements of *RGS1* and *CREB5*. HEK 293 cells were transfected with the indicated reporter genes and expression vectors. Myc‐ZNF384/pSG5, Flag‐EP300‐Z/pCMV, Flag‐TCF3‐Z/pcDNA, and Flag‐TAF15‐Z/pcDNA were used as the expression vectors of ZNF384 and Z‐fusion proteins. A luciferase assay was performed 48 h after transfection. Relative luciferase activities are plotted on bar charts as the average values of three independent experiments. Error bars indicate SEM.

Expressions of the six candidate genes were confirmed by RT‐qPCR. The expressions of all six genes were significantly higher in Z‐fusion‐expressing LCL than control LCL, which was consistent with the results of RNA‐seq (Figure 3A). We found binding regions of ZNF384 in these genes using ENCODE ChIP‐seq data (Supplemental Figure S1), and performed ChIP‐qPCR to examine whether Z‐fusion proteins commonly bound to these genes in LCL transfectants. TCF3‐Z bound to all six genes, while TAF15‐Z bound to *CREB5* and *RGS1*, (Figure 3B). These results indicate that *CREB5* and *RGS1* were the direct transcriptional targets of both Z‐fusion proteins.

For further confirmation of the direct upregulation of *RGS1* and *CREB5* by Z‐fusion proteins, we evaluated the expression of a luciferase reporter gene placed downstream of the promoter elements of *RGS1* and *CREB5*. The expression of Z‐fusion proteins strongly increased luciferase expression, indicating the direct transcriptional activity of Z‐fusion proteins on them (Figure 3C).

We further confirmed the upregulation of *RGS1* and *CREB5* by Z‐fusion proteins in another B‐cell cell line. Introduction of the expression vectors of Z‐fusion proteins into NALM‐6 caused increased expression of the mRNA of *RGS1* and *CREB5* (Supplemental Figure S2).

### 3.4 Knockdown of CREB5 and RGS1 did not affect the growth of Z‐fusion‐positive ALL cells

**FIGURE 4:**
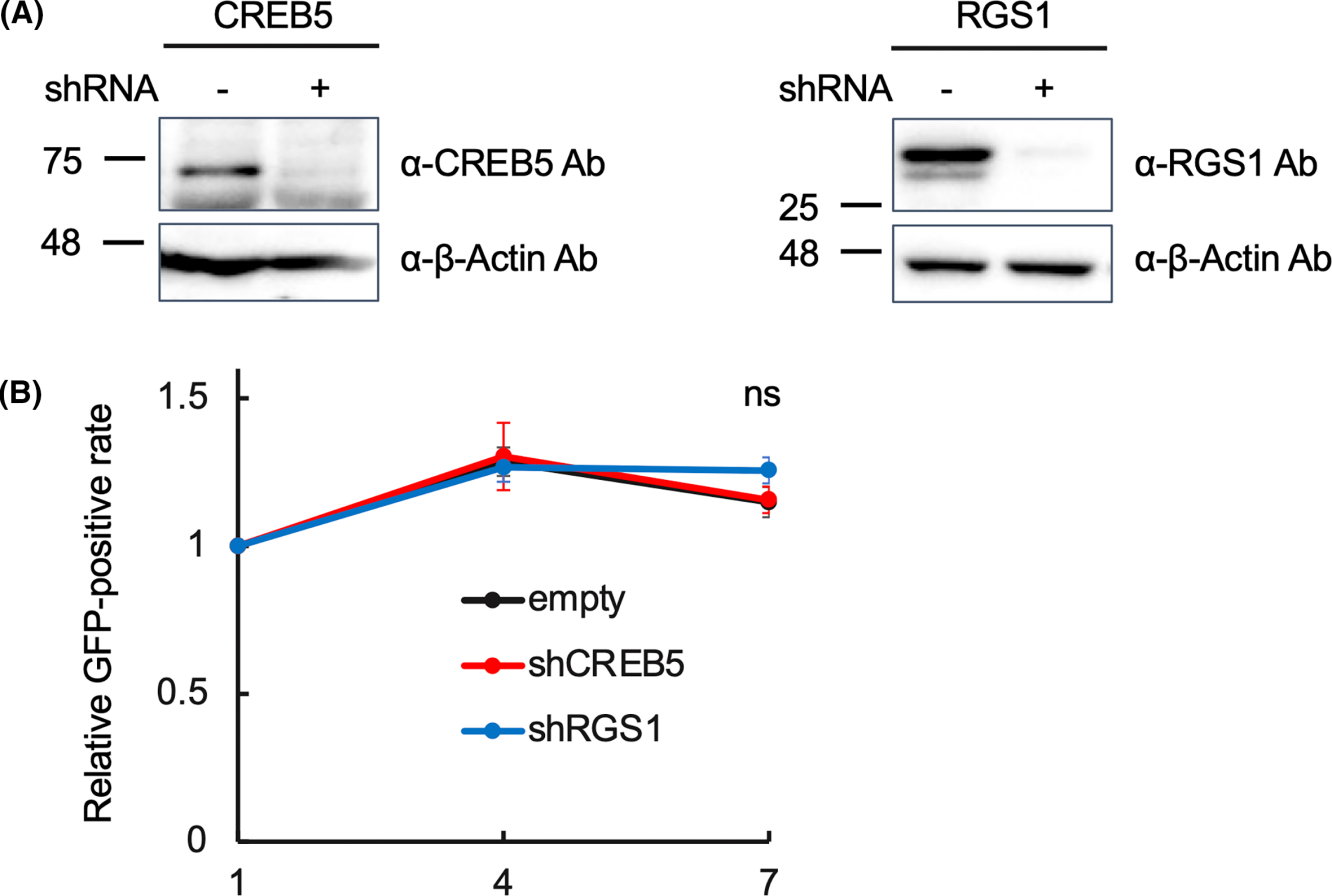
Knockdown of CREB5 and RGS1 did not affect the growth of Z‐fusion‐positive ALL cells. (A) The effects of shRNA against CREB5 and RGS1. The expression vectors of CREB5 (left panel) and RGS1 (right panel) were co‐expressed with the expression vectors of the indicated shRNA in HEK293T. The abilities of these shRNA to knockdown the target genes were confirmed by immunoblots with the indicated antibodies 48 h after transfection. (B) Growth of JIH5 after the knockdown of CREB5 or RGS1. The indicated expression vectors of shRNA were introduced into JIH5 by electroporation. The rate of GFP‐positive cells was analyzed by frow cytometry at the indicated time points, and relative GFP‐positivity was plotted on line charts as the average value of three independent experiments. Error bars indicate SEM. Statistical analyses were done as in Figure 3A.

In order to examine whether CREB5 and RGS1 affected the growth rate of Z‐fusion protein‐expressing cells, we investigated the growth rates of LCL transfectants; however, the expression of Z‐fusion proteins did not significantly affect them (Supplemental Figure S3). Next, we conducted a knockdown experiment to investigate the contribution of CREB5 and RGS1 to growth of the Z‐fusion (+) ALL cell line, JIH5. We cotransfected HEK293T with expression vectors of CREB5 and RGS1 and shRNA expression vectors against them by lipofection to confirm the knockdown effect of the shRNA, because no available antibody could detect endogenous expressions of CREB5 and RGS1 in JIH5 or HEK293T. Successful knockdown was confirmed by immunoblot (Figure 4A). Then, we introduced shRNA vectors into JIH5 by electroporation. GFP was used as a marker of the gene introductions, and the efficiency of these introductions ranged from 30% to 40%. Time‐course analyses of the positive rate of GFP revealed that the growth rates of shRNA vector‐introduced cells were similar to those of empty vector‐introduced cells (Figure 4B), indicating that CREB5 and RGS1 did not significantly contribute to the growth of JIH5.

### 3.5 Z‐fusion protein‐induced upregulation of RGS1 impaired CXCL12‐induced cell migration

**FIGURE 5:**
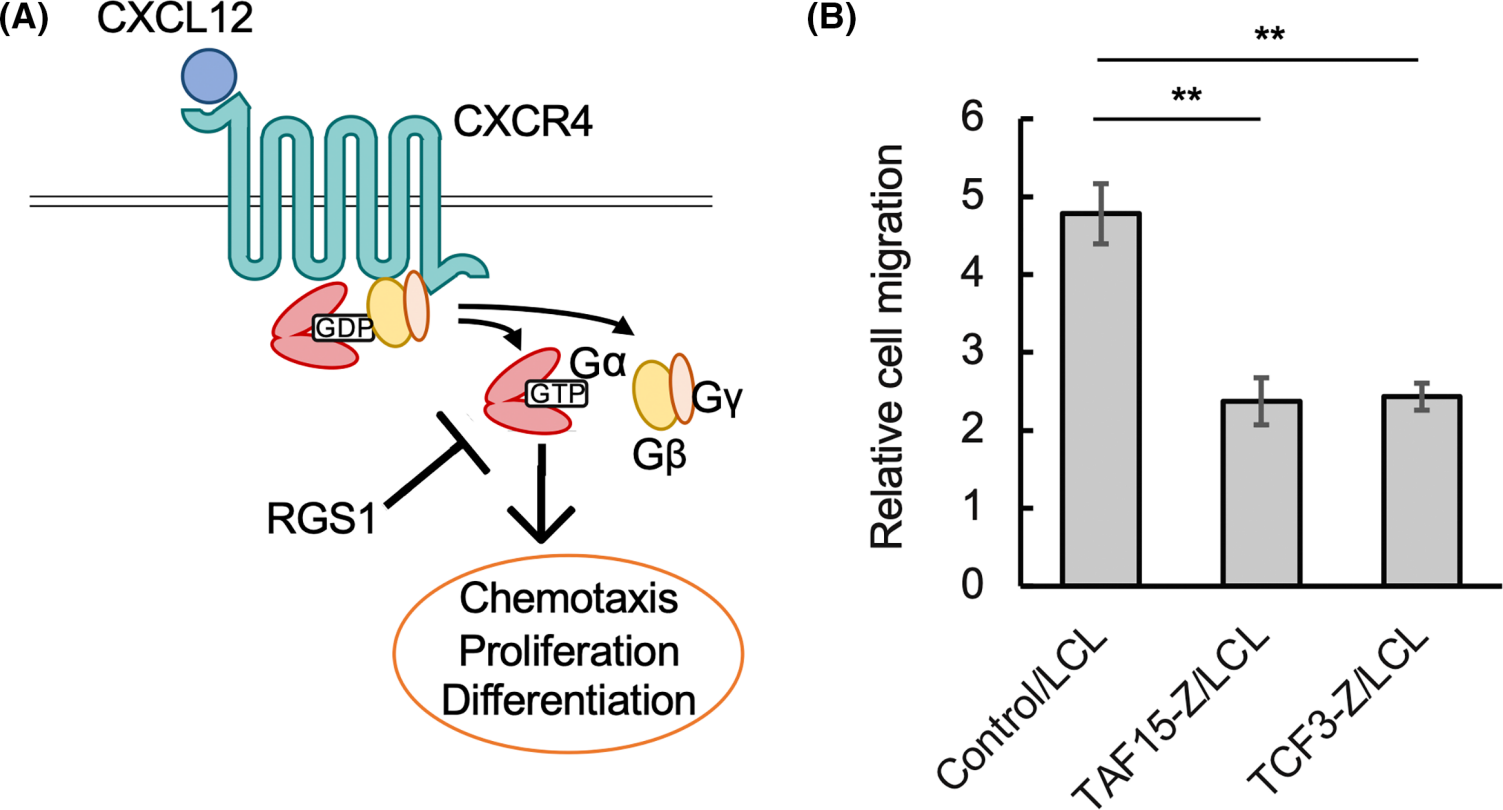
Z‐fusion protein‐expressing LCL cells showed impaired migration to CXCL12. (A) Schematic presentation of the role of RGS1 in CXCL12‐CXCR4 signaling. After CXCL12 binds to CXCR4, the Gαβγ heterotrimer binds to CXCR4, which exchanges GDP on the Gα subunit for GTP, and this results in dissociation of the complex to Gα and Gβγ subunits. The Gα and Gβγ subunits activate downstream signaling pathways. RGS1 binds to Gα–GTP, accelerates the rate of GTP hydrolysis to GDP, and terminates the signal. (B) Cell migration assay. The indicated LCL transfectants were treated with 1 μg/mL Dox for 2 days and serum‐starved for 8 h prior to the assay. Then, 2 × 10^5^ cells were seeded on the upper chambers in serum‐free medium. The media in the lower chambers were serum‐free with or without supplementation of 100 ng/mL CXCL12. After 24 h, cells that had migrated into lower chambers were measured. Numbers of cells that had migrated to the lower chambers with CXCL12 supplementation relative to that without CXCL12 supplementation were plotted on bar charts as the average values of three independent experiments. Error bars indicate SEM. Statistical analyses were done as in Figure 3A.

It is known that RGS1 works as a specific inhibitor of CXCR4 signaling through the inhibition of G‐protein activated by CXCR4. CXCR4 is a chemokine receptor. Binding of CXCL12, a ligand for CXCR4, to CXCR4 activates CXCR4 signaling and causes chemotaxis toward CXCL12, proliferation, and differentiation (Figure 5A).^24^ Therefore, we examined whether Z‐fusion protein‐induced RGS1 expression affected chemotaxis toward CXCL12 by the transwell assay. Cell migrations toward CXCL12 were significantly impaired in both TCF3‐Z/LCL and TAF15‐Z/LCL compared with Control/LCL (Figure 5B), indicating that Z‐fusion protein‐induced RGS1 expression could inhibit CXCL12‐CXCR4 signaling.

## 4 DISCUSSION

In the present study, we screened for both DEG in cell line transfectants and one in clinical samples to search for transcriptional targets of Z‐fusion proteins, and identified *CREB5* and *RGS1* as direct and common transcriptional targets of Z‐fusion proteins. There are two reported studies searching for transcriptional targets of Z‐fusion proteins. One screened for DEG in clinical samples comparing Z‐fusion (+) ALL and other ALL, and selected *cardiotrophin‐like‐cytokine factor 1* (*CLCF1*) and *B‐ and T‐lymphocyte attenuator* (*BTLA*)^6^; however, the ectopic expression of Z‐fusion proteins did not upregulate both genes very strongly, log_2_Fold Change <1, in our study and that by others.^21^ The other screened for DEG in Reh, an *ETV6::RUNX1*‐positive ALL cell line, comparing EP300‐Z‐transfected Reh and control Reh, and selected *GATA3* ^21^; however, *GATA3* was not upregulated in TCF3‐Z/LCL or TAF15‐Z/LCL in our study, probably due to the difference of the Z‐fusion gene used. No model was proposed concerning how upregulation of these genes contributes to ALL development. There will be a common mechanism, common transcriptional target genes whose upregulation contributes to ALL development, among various Z‐fusion proteins, because almost all Z‐fusion (+) ALL demonstrated similar gene expression profiles despite a wide variety of fusion partner genes.^6^, ^8^ If so, such a target gene should be upregulated in transfectants of various Z‐fusion genes and primary ALL cells with various Z‐fusion genes. Thus, we screened for both DEG in cell line transfectants and clinical samples.

In the screening using LCL, we compared Z‐fusion transfectants to empty‐vector transfectant, not to ZNF384‐transfectant, because using it as a control might mask important gene expression changes for oncogenesis. It is reported that overexpression of ZNF384 has oncogenic potential for some solid tumors, such as hepatocellular carcinoma and breast cancer.^22^, ^23^ Because Z‐fusion proteins contain all of ZNF384, it is expected that the DNA binding sites are quite similar between them.

Several studies focused on FLT3 as a Z‐fusion protein‐specific transcriptional target.^14^, ^24^ Zhao et al. focused on FLT3 due to its higher expression in Z‐fusion (+) ALL than other ALL, and identified Z‐fusion protein‐specific‐binding to FLT3 enhancer in patient‐derived xenograft cells of Z‐fusion gene‐positive ALL.^24^ Dickerson et al. focused on FLT3 based on the results of GREAT analysis using the set of the genes that Z‐fusion proteins bound to more strongly than ZNF384 in ChIP‐seq as a data set. They showed that FLT3 expression was higher in Z‐fusion gene‐transduced mouse pre‐B cells than in ZNF384‐transduced ones.^14^ These findings indicate that FLT3 is one of the common and direct targets of Z‐fusion proteins. In the present study, FLT3 was included in DEG in clinical samples (Table S3) but not in LCL transfectatnts (Table S2). The induction of FLT3 by Z‐fusion proteins was insufficient in LCL transfectants (log_2_FC = 0.7), and its expression level was low (baseMean = 10). Thus, FLT3 was not selected by our screening. Previous studies selected FLT3 based on its reported biological significance in some screening procedures, which may be the reason for the different results.

RGS1 belongs to the regulator of G‐protein signaling (RGS) family and regulates G‐protein signaling, as its name suggests. G‐protein is a downstream effector of G‐protein coupled receptor (GPCR). RGS family proteins inactivate G‐protein and inhibit GPCR signaling (Figure 5A).^25^ RGS1 has been shown to inactivate CXCL12‐CXCR4 signaling in B cells. Overexpression of RGS1 in pro‐B cells impaired CXCL12‐induced chemotaxis and adhesion to VCAM‐1.^26^ Furthermore, the CXCL12‐CXCR4 axis is important for the homing and retention of hematopoietic progenitor cells in the marrow microenvironment. B‐cell development was severely impaired in CXCL12 conditional knockout mice, probably due to the lack of B‐cell‐differentiating stimulation from the marrow microenvironment.^27^ These findings suggest that RGS1 overexpression by Z‐fusion proteins impairs B‐cell differentiation by inhibiting the CXCL12‐CXCR4 axis. Although we demonstrated that Z‐fusion‐induced RGS1 expression was sufficient for the inhibition of cell migration to CXCL12 in vitro, in vivo analysis is required to examine whether Z‐fusion‐induction causes impaired homing and retention in the marrow microenvironment.

The concept whereby inhibition of the CXCL12‐CXCR4 axis by RGS1 contributes to leukemogenesis might appear to contradict recent findings showing that leukemia cells use the CXCL12‐CXCR4 axis to access and reside in a bone marrow microenvironment that is expected to favor their growth and survival.^28^ However, it was also reported in a study using Cxcl12 conditional knockout mice that the loss of Cxcl12 conferred a survival advantage to hematopoietic progenitors and promoted faster hematologic recovery after 5‐fluorouracil‐induced myelosuppression due to the loss of quiescence of progenitors.^27^ These findings suggest that at least short‐term hematopoiesis is independent of the CXCL12‐CXCR4 axis. If so, it is possible that some types of leukemia develop independently of the CXCL12‐CXCR4 axis.

CREB5 is a transcription factor belonging to the cyclic AMP response element binding protein (CREB) family. The CREB family upregulates genes involved in cell‐cycle acceleration and cell proliferation. Transgenic mice of CREB1 developed myeloproliferative disease,^29^ while knockdown of CREB1 impaired proliferation of HSC and AML cells,^30^ indicating the critical role of CREB1 in myeloid cell proliferation. Although the detailed function of CREB5 has yet to be clarified, it has been reported that CREB5 is highly expressed in several cancers, such as colorectal cancer, epithelial ovarian cancer, and hepatocellular carcinoma, and involved in their metastasis, invasion, and proliferation.^31^, ^32^, ^33^ These findings suggest that CREB5 contributes to ALL development by enhancing cell proliferation; however, a knockdown experiment revealed that CREB5 did not significantly contribute to the growth of JIH5 (Figure 4B). One possible explanation for this contradiction is that the growth dependency on CREB5 has been abolished in JIH5 by additional gene abnormalities acquired during the leukemia development or transformation of primary leukemia cells to the cell line. It was reported that JIH5 was established from primary ALL cells at the second relapse and there were additional somatic mutations in 8 genes and 4 additional fusion genes.^34^ The contribution of CREB5 to leukemia cell growth should be investigated using primary Z‐fusion (+) ALL cells.

We could not show substantial evidence supporting the notion that CREB5 and RGS1 contribute to leukemogenesis by Z‐fusion proteins. The mechanism of leukemogenesis by Z‐fusion proteins remains poorly understood. Further studies are required to confirm the contribution of RGS1 and CREB5 to ALL development, such as an investigation of whether knockdown of these genes prevents Z‐fusion gene‐induced ALL development in a mouse model.

## AUTHOR CONTRIBUTIONS

**Chiharu Yamada:** Data curation (supporting); formal analysis (supporting); investigation (lead); methodology (supporting); project administration (supporting); validation (lead); writing – original draft (supporting). **Kentaro Okada:** Data curation (supporting); formal analysis (supporting); investigation (lead); methodology (supporting); project administration (supporting); writing – original draft (supporting). **Koya Odaira:** Formal analysis (supporting); investigation (supporting); methodology (supporting); writing – review and editing (supporting). **Mahiru Tokoro:** Formal analysis (supporting); writing – review and editing (supporting). **Eisuke Iwamoto:** Data curation (supporting); formal analysis (supporting); writing – review and editing (supporting). **Masashi Sanada:** Data curation (supporting); formal analysis (supporting); writing – review and editing (supporting). **Mina Noura:** Data curation (supporting); formal analysis (supporting); investigation (supporting); methodology (supporting); writing – review and editing (supporting). **Syuichi Okamoto:** Methodology (supporting); writing – review and editing (supporting). **Takahiko Yasuda:** Data curation (lead); formal analysis (lead); investigation (supporting); writing – review and editing (supporting). **Shinobu Tsuzuki:** Methodology (supporting); writing – review and editing (supporting). **Hitoshi Kiyoi:** Methodology (supporting); writing – review and editing (supporting). **Fumihiko Hayakawa:** Conceptualization (lead); data curation (lead); formal analysis (lead); funding acquisition (lead); investigation (supporting); methodology (lead); project administration (lead); resources (lead); supervision (lead); writing – original draft (lead).

## FUNDING INFORMATION

This work was supported by JSPS KAKENHI grant numbers 18H02835, 21 K19505, and 22H03102 (to F.H.); Grants for Practical Research for Innovative Cancer Control grant numbers JP19ck0106331, JP22ck0106607, and JP 23ck0106851 (to F.H.) from the Japan Agency for Medical Research and Development (AMED). We sincerely thank all for their support.

## CONFLICT OF INTEREST STATEMENT

All authors state that they have no potential competing interests in relation to this study. H. Kiyoi is an Editorial Board Member of Cancer Science.

## ETHICS STATEMENT

Approval of the research protocol by an Institutional Reviewer Board: Yes.

The ethics committee of Nagoya University Graduate School of Medicine.

Informed Consent: N/A.

Registry and the Registration No. of the study/trial: N/A.

Animal Studies: N/A.

## Supporting information


Figure S1.

Table S1.

## Data Availability

The data that support the findings of this study are available from the corresponding author upon reasonable request.
