# Supplementary material for: RGS1 and CREB5 are direct and common transcriptional targets of ZNF384‐fusion proteins
Source: Cancer Med. 2024 Jul 17;13(14):e7471. doi: 10.1002/cam4.7471 (PMC11252495; doi:10.1002/cam4.7471)
Supplement: Supplementary file 1 — Figure S1. [file CAM4-13-e7471-s002.docx]

**Supplemental Figure S1. Expected Z-fusion protein-binding regions near candidate target genes from ChIP-seq data**

Data on ZNF384 binding around the indicated genes were obtained from the Encode ChIP-seq data library and are demonstrated. The amplified regions in ChIP-qPCR are indicated by black arrows. Primers are shown in Table 1.

**Supplemental Figure S2. Upregulations of *RGS1* and *CREB5* by Z-fusion gene introduction in B-ALL cell line**

The expression vectors of the indicated genes were introduced into NALM-6 by electroporation. mRNA was extracted from the indicated cells after 48 h and subjected to RT-qPCR. Expression levels of the indicated genes are plotted on bar charts as values relative to *GAPDH* expression. Experiments were independently performed three times in duplicate and average values were plotted. Error bars indicate SEM. Welch’s t-test was used for the statistical test. *: P < 0.05; **:P<0.01.

**Supplemental Figure S3. Expression of Z-fusion proteins did not significantly affect the growth rate of LCL.**

The indicated cells were cultured with 1 µg/mL Dox for 48 h. Cell proliferations were measured every 24 h by WST-8 assay. WST-8 values relative to that on Day 0 are plotted on line charts with their standard errors (SEM).
